# The Georgia State Dental Board

**Published:** 1886-06

**Authors:** 


THE GEORGIA STATE DENTAL BOARD.—"*University of Maryland, Faculty of Physic, Department of Dental Surgery, Baltimore, May 20th, 1886.*—The Secretary of the Georgia State Board of Dental Examiners, Dr. L. D. Carpenter, of Atlanta, Georgia, having submitted the questions on Therapeutics, Operative Dentistry, Pathology, Physiology, Anatomy, Chemistry, Materia Medica, Prosthetic Dentistry and Metallurgy, which were presented to the applicants for license to practice dentistry in the State of Georgia at the recent examination held by the State Board of Dental Examiners, to the undersigned, members of the Faculty of the *Dental Department of the University of Maryland*, for their opinion of said questions, we desire to state that we have carefully examined the same, and consider them to be well-selected, fair and equitable, and such as all graduates of dental schools, and more especially recent graduates, should be able to answer satisfactorily and successfully.

Ferdinand J. S. Gorgas, M. D., D. D. S.,
**Professor of Principles of Dental Science, Dental Surgery, Dental Prosthesis and Dental Materia Medica.**

James H. Harris, M. D., D. D. S.,
**Professor of Operative and Clinical Dentistry.**

Francis T. Miles, M. D.,
**Professor of Physiology.**

J. Edwin Michael, M. D.,
**Professor of Anatomy.**

R. Dorsey Coale,
**Professor of Chemistry and Metaliurgy.**

